# Lymphatic filariasis: Aspiration of adult gravid female worm from a soft tissue swelling

**DOI:** 10.4103/0970-9371.73308

**Published:** 2010-10

**Authors:** Kalpana Azad, Rashmi Arora, Kusum Gupta, Uma Sharma

**Affiliations:** Department of Pathology, V.M.M.C. and Safdarjung Hospital, New Delhi, India

Sir,

A 35 year-old male resident of Bihar, presented with a soft tissue swelling along the medial aspect of right arm, measuring 2 cm in diameter, of 4 months duration, associated with local pain. He had low grade fever since 2 months and his routine hemogram was normal. His erythrocyte sedimentation rate (ESR) was 64 mm at the end of 1 hour. Clinical diagnosis of tuberculosis was made.

**Figure 1:**
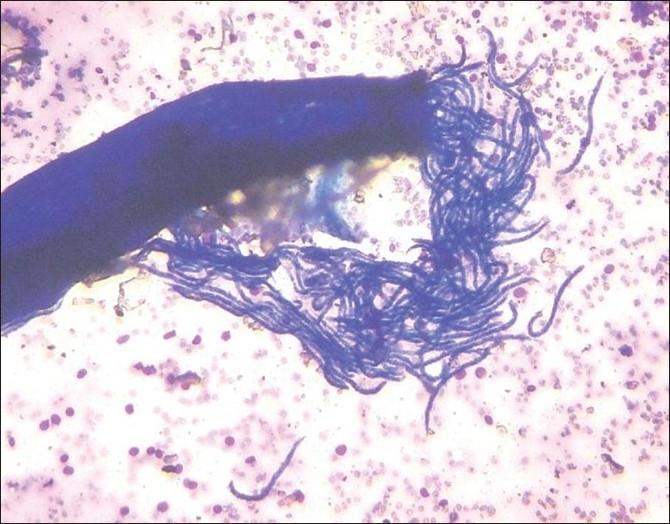
Adult gravid female worm with numerous microfilariae (Giemsa, ×40)

**Figure 2:**
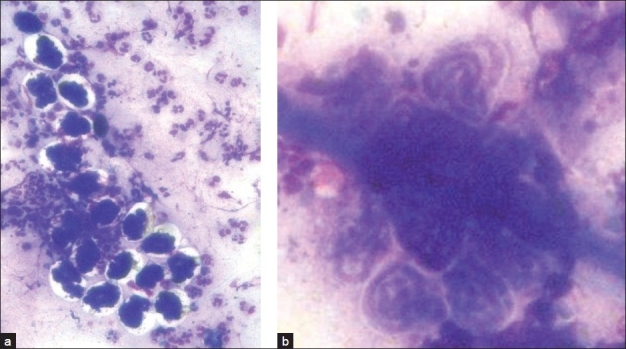
(a) Embryonated eggs and coiled microfilariae (Giemsa, ×100), (b) coiled microfilariae (Giemsa, ×100)

Fine needle aspiration cytology (FNAC) from the nodule yielded fluid along with a creamy white thread. Smears showed adult gravid female filarial worm having an intact outer cuticle layer and body cavity containing paired uteri filled with different stages of developing microfilariae. Also, few microfilariae were seen protruding out from the breach in the cuticle layer [Figure 1]. In addition, numerous embryos and coiled larvae [Figure 2] and fully straightened larvae of *Wuchereria bancrofti* which were sheathed and had no nuclei in the tail end were seen. The background was composed of inflammatory cells including neutrophils, lymphocytes, macrophages and eosinophils.

The patient was then treated with oral diethylcarbamazine and his swelling disappeared. He was followed for 4 months and is doing well.

Filariasis is a major health problem in tropical countries including the Indian subcontinent. It is caused by any of three closely related parasitic nematodes - *Wuchereria bancrofti, Brugia malayi* or *Brugia timori*.

*Wuchereria bancrofti*, a human nematode parasite, is capable of producing disease due to migration of adult parasite through the lymphatic system.[1] The adult *Wuchereria bancrofti* may produce lesions by involving the lymphatics of the lower limbs, spermatic cord, epididymis, testis, retroperitoneum and female breast.[1] Its typical presentation are elephantiasis, chronic lymphoedema, epididymitis, funiculitis and lymphadenitis.[1,2] In spite of a large number of such lesions diagnosed on cytology, it is unusual to find adult filarial worms.

Most people with microfilaraemia do not show signs or symptoms of the disease but are important source of infection in the community. Symptomatic lymphatic filariasis has very low microfilaraemia. Thus, disease and infection do not necessarily accompany each other.[3]

In the present case, the patient had a solitary swelling from which fluid and fragment of adult worm was aspirated. There are a few reports of adult filarial worms in aspirates of soft tissue swellings,[1,3] lymph node[4,5] and epididymal nodules. In all these cases, the swelling was painless and the patient was asymptomatic. In none of the cases, a clinical diagnosis of filariasis was considered. Most of the soft tissue swellings associated with fever are diagnosed as tuberculous or pyogenic. FNAC can distinguish between these swellings accurately and plays an important role in their correct diagnosis.

Thus, in countries where lymphatic filariasis is endemic, it should be considered in the clinical differential diagnosis of a soft tissue swelling. FNAC is a convenient and effective diagnostic method in patients with soft tissue mass. Thus, to conclude, identification of parasite in cytology smears plays an important role in diagnosis of disease and institution of specific treatment.
